# Variant *Plasmodium ovale *isolated from a patient infected in Ghana

**DOI:** 10.1186/1475-2875-10-15

**Published:** 2011-01-22

**Authors:** David Tordrup, Jakob Virenfeldt, Felicie F Andersen, Eskild Petersen

**Affiliations:** 1 Department of Molecular Biology, Faculty of Science, University of Aarhus, Aarhus, Denmark; 2 Department of Infectious Diseases, Institute of Clinical Medicine, Aarhus University Hospital-Skejby, DK-8200 Aarhus N., Denmark

## Abstract

Recent data have found that *Plasmodium ovale *can be separated in two distinct species: classic and variant *P. ovale *based on multilocus typing of different genes. This study presents a *P. ovale *isolate from a patient infected in Ghana together with an analysis of the small subunit RNA, cytochrome b, cytochrome c oxidase I, cysteine protease and lactate dehydrogenase genes, which show that the sample is a variant *P. ovale *and identical or highly similar to variant *P. ovale *isolated from humans in South-East Asia and Africa, and from a chimpanzee in Cameroon. The split between the variant and classic *P. ovale *is estimated to have occurred 1.7 million years ago.

## Background

It is usually assumed that the four major human malaria species *Plasmodium falciparum, Plasmodium vivax, Plasmodium ovale *and *Plasmodium malariae *have no animal reservoirs and that zoonotic *Plasmodium *infections in humans are highly unusual. The recent finding that *Plasmodium knowlesi *is found in several countries in Southeast Asia, where it has been wrongly classified as *P. malariae*, shows that molecular typing may change the understanding of human malaria as an infection strictly transmitted between humans[1,2]. *Plasmodium ovale *was first described from West Africa in 1922 and subsequently from every continent[3]. In West Africa, a blood film *P. ovale *parasite positive rate between 0.7% and 10% has been found[4]. However, in other areas the occurrence of isolated cases is difficult to explain without an animal reservoir[3].

Characterization of *P. ovale *from Southeast Asia based on the small subunit rRNA gene and parts of the *cysteine protease, ookinete surface protein *and *cytochrome b *genes indicate that *P. ovale *can be divided into at least two types, classic and variant, which do not differ morphologically[5,6]. Variant *P. ovale *seems to be associated with higher parasite density in humans[7-9]. A recent study of 55 *P. ovale *isolates from around the world clearly showed that variant and classic *P. ovale *co-exist and do not recombine[10].

A study comparing sequences from the *cytochrome b, cytochrome c oxidase 1 *and *lactate dehydrogenase *genes of *Plasmodium *spp. from gorilla (*Gorilla gorilla*) and chimpanzees (*Pan troglodytes*) with *P. ovale *from humans found one variant *P. ovale *in a chimpanzee closely related to the human variant[11].

This study describes the molecular characterization of a *P. ovale *isolate from a patient infected in Ghana, showing that the isolate is a variant *P. ovale *based on the characterization of five genes, and that it is identical or highly similar to a *P. ovale *variant isolated from a chimpanzee[11] and from humans in two separate studies[5,10]. The time of the phylogenetic split between the two species is estimated at 1,7 million years ago.

## Methods

### Patient

The patient was a 59-year-old Danish male, admitted with fever in March 2009 after traveling in Ghana for two weeks in November 2008, who had not taken prophylaxis regularly. The itinerary included Accra, the Volta River and a resort on the Cape Coast.

Except for hypertension and atrial fibrillation, he was previously healthy. He had visited Ghana for short visits in 2005, 06 and 07. Fever started nine days before admission, with no focal symptoms. Physical examination at admission was normal. At admission the laboratory tests showed a C-reactive protein of 59.9 mg/l (< 8), haemoglobin 9.4 mmol/lit (8.1 - 10.3), white blood cell count of 6.0 with relative lymphopenia of 0.21 (1.3 - 3.5 x 10^9/l) and a thrombocytopaenia of 93 (145 - 350 x 10^9/l).

**Figure 1:**
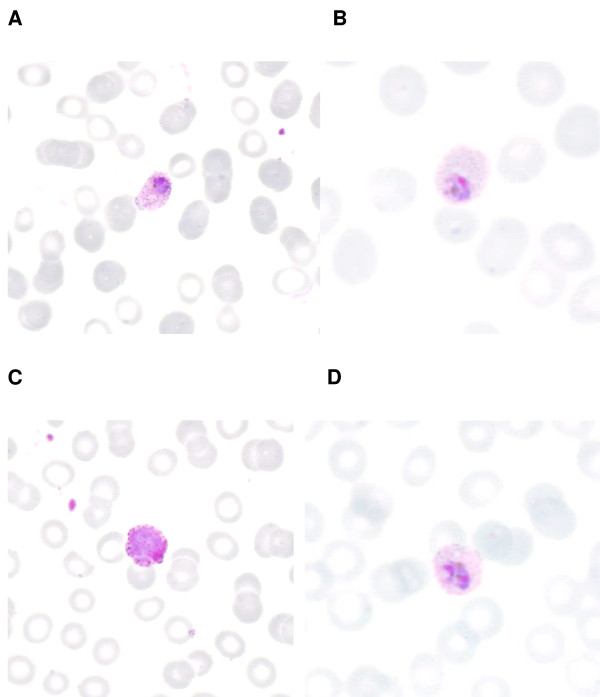
Giemsa stained, thin blood film showing *P. ovale* throphozoite with Schüffner dots (A and B); a *P.ovale* gametocyte (C); and an early schizont with two chromatin dots (D).

Thick and thin blood films showed a *P. ovale *infection with an initial parasitaemia of 0.5% and numeration of the parasite density found 11,600 parasites per μl blood (Figure 1). The second day the parasitaemia was estimated to 0.1% with a density of 1,100 parasites per μl blood. Quick-test (Binax, Bedford, UK) was negative. Chest X-ray showed pneumonia on the left side, and the patient was treated with ceftriaxone intravenously.

The patient was treated with chloroquine phosphate over three days followed by primaquine 15 mg daily for 2 weeks. During the first two days hypotension with a systolic pressure of 80 mmHg was successfully treated with infusions of saline. The patient was discharged on day 7 in good condition and no relapse has been observed over an eighteen month follow up.

### Isolation of parasitic genomic DNA and PCR

Parasitic genomic DNA was extracted from whole blood using NucleoSpin Blood QuickPure (Macherey-Nagel, Düren, Germany) according to manufacturer's protocol. All PCR reactions were performed with Expand High Fidelity PCR System (Roche, Basel, Switzerland) using primers and concentrations as described in Additional file 1: table S1. Reactions also contained 5 μL 1/100 diluted genomic DNA (*possrdna*) or 1uL 1/10 diluted DNA (all other genes), 200 μM each dNTP, 2,6U enzyme (1U for *poldh *and *possrnda *inner nests) and 1,5 mM MgCl2 in Expand High Fidelity Buffer, final volume 50 μL (20 μL for inner nest reactions).

### Cloning of PCR products

The PCR products were cloned into either the pYES2.1/V5-His-TOPO vector (Invitrogen, Carlsbad, California) and progated in *E. coli *Top10 cells (Invitrogen, Carlsbad, California) (*possrdna, pocytb*) or cloned into the pcDNA2.1/V5-His-TOPO vector (Invitrogen, Carlsbad, California) and propagated in XL1-Blue cells (*pocox1, poldh, pocysp*) under ampicillin selection

### Plasmid purification and sequencing

Plasmids were purified using Qiagen Plasmid Mini Kit (Qiagen, Hilden, Germany) according to manufacturers protocol. Sequencing was performed with BigDye Terminator 3.1 (Applied Biosystems, Foster City, California) on an ABI3130 platform. Three separate clones from each construct (except *pocysp *for which only one clone was sequenced) were sequenced; conflicts were resolved by majority vote.

### Sequence alignments and phylogenetic analyses

Sequencing data was assembled and consensus sequences aligned in CLC Main Workbench 5.6 (CLC bio, Aarhus, Denmark), gap open cost = 10, gap extension cost = 1 and end gap cost = as any other. Phylogenetic trees were calculated in CLC Main Workbench 5.6 using Maximum Likelihood Phylogeny. The initial tree was calculated by Neighbor Joining and a HKY substitution model with a transition/transversion ratio of 2.0 was used for inferring phylogenetic relationships.

### Estimation of divergence time

The sequences of *pocytb *and *pocox1 *from the patient isolate were concatenated in CLC Main Workbench 5.6 and the resulting sequence was aligned to concatenated *pocytb *and *pocox1 *sequences from 11 *Plasmodium *species in NCBI GenBank (for classic *P. ovale *FJ409567 and FJ409571 were used for *pocytb *and *pocox1 *respectively). The final alignment was 1671 nucleotides in length. The BEAST software[12] was used to estimate the divergence time, using *Plasmodium gallinaceum *as an outgroup and assuming a divergence time of 6 +/- 0.5 MY (normal distribution) for *P. falciparum/Plasmodium reichenowi *for calibration of the tree. The site model was HKY with estimated base frequencies and a 4 category gamma site heterogeneity model. An uncorrelated lognormal relaxed clock was used, and the tree prior was set to Yule Process. The chain was run for 2.000.000 cycles and data points were logged at every 200 cycles.

## Results

Fever developed four months after a two-week visit to Ghana and at admission, the patient had a microscopically diagnosed *P. ovale *infection with a parasitaemia of 0.5% and the presence of asexual forms, early schizonts and gametocytes (Figure 1). A rapid test (Binax^®^) was negative. Because of the recent reports of variants of *P. ovale *and because the patient had a negative rapid test, the isolate was sequenced for further characterization.

Part of the asexually transcribed small subunit (18S) ribosomal DNA (*possrdna*) gene from the isolate was compared with classic and variant sequences published in recent studies from Asia and Africa[5,10], revealing closer resemblance to the variant type from both studies (table 1). To further characterize the isolate, the *pocytb, pocysp, pocox1 and poldh *genes were sequenced and compared with sequences from three recent studies. The isolate sequences were 100% identical to the *pocytb *variant type sequences reported in all three studies[5,10,11], 99,8% identical to variant type *cysp *from Asian isolates[5], and 100% identical to variant type *pocox1 *and *poldh *from African isolates[11]. The isolate was also compared to *pocytb, pocox1 *and *poldh *sequences isolated from a chimpanzee[11], revealing 100% identity in all three genes (Table 1).

**Table 1:** Comparison of the present isolate with sequences published by Sutherland *et al *[10], Win *et al *[5] and Duval *et al *[11].

	*possrdna*	*pocytb*	*pocysp*	*poldh*	*pocox1*
**Variant**					

Win et al.	1181-1187/1192-1199 (98,8-99,5%)	708/708 (100%)	530/531 (99,8%)	No data	No data

Sutherland et al.	715-719/722-727 (98,8-99,3%)	708/708 (100%)	No data	No data	No data

Duval et al.	No data	708/708 (100%)	No data	351/351 (100%)	963/963 (100%)

**Chimpanzee**					

Duval et al.	No data	708/708 (100%)	No data	351/351 (100%)	963/963 (100%)

**Classic**					

Win et al.	1160-1162/1192-1199 (96,8-96,9%)	698/708 (98,6%)	512/531 (96,4%)	No data	No data

Sutherland et al.	690-707/722-727 (95,4-97,7%)	698/708 (98,6%)	No data	No data	No data

Duval et al.	No data	698/708 (98,6%)	No data	338/351 (96,3%)	953/963 (99,0%)

Comparisons are given as identities/sequence length (percent), except for *possrna *which are given as a range of identities/range of sequence lengths (percent range) due to varying lengths and polymorphisms in this gene.

**Figure 2:**
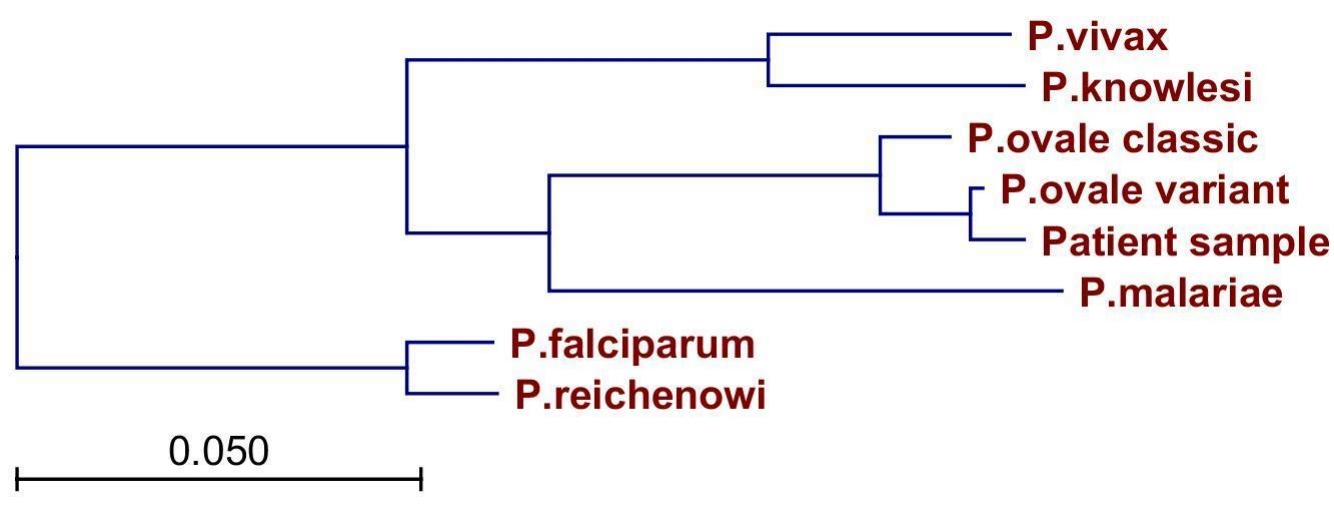
**Phylogenetic tree based on the concatenated sequences of *possrdna, poldh, pocysp, pocox1 *and *pocytb***. Scale bar indicates expected number of substitutions per nucleotide site.

A phylogenetic tree was constructed based on the concatenated sequences of all five genes studied and sequences published in GenBank (Figure 2). The *P. ovale *variant type together with the present isolate formed a clade related to but separate from the classic type. Calculation of phylogenetic trees based on individual genes yielded equivalent results. To estimate the split between variant and classic *P. ovale*, Bayesian Inference was used as implemented in the BEAST software[12] with a calibration point of 6 Million Years (MYs) +/- 0.5 MYs for the human/chimpanzee speciation event as represented by the *P. falciparum/P. reichenowi *split, which gave an estimated age of 1.7 MY (95% Highest Posterior Density 0.25-4.44MY) for the most recent common ancestor of the variant and classic *P. ovale*.

## Discussion

The recent study of 55 *P. ovale *isolates from around the world clearly showed that *P. ovale *can be divided in two species: classic and variant (suggested named *Plasmodium ovale curtisi *and *Plasmodium ovale wallikeri*) [10]. The two species currently circulate side by side in the same geographical areas and are not recombining even though they infect the same host. It is not known whether variant *P. ovale *continues to circulate in both humans and primates and it is intriguing that the variant and classic strains do not recombine. One explanation for the speciation could be specificity for different *Anopheles *species[10,13], another possibility is receptor divergence. Humans ceased producing N-glycolylneuraminic acid (Neu5Gc) around 2 to 3 million years ago but continued producing N-acetylneuraminic acid (Neu5Ac) in contrast to old-world primates who synthesize both[14]. Neu5Gc has been shown to interfere with the binding of *P. falciparum *Erythrocyte-Binding Antigen 175 (PfEBL-175) to chimpanzee erythrocytes, possibly explaining why *P. falciparum *does not infect chimpanzees[15]. The evolutionary timing of the loss of Neu5Gc coincides with our estimated divergence time of 1.7 million years, which is in agreement with the estimate of Sutherland et al. [10], and could explain the split between the two *P. ovale *variants by a similar mechanism in which only classic *P. ovale *is sensitive to Neu5Gc interference.

The chimpanzee can be infected with *P. ovale*, but the infection is self-limiting[16,17], though it is not known whether these experiments were performed with variant or classic *P. ovale. P. ovale *in chimpanzees closely related or identical to human variant *P. ovale *has been described in Cameroon[11], and three different genes from the isolate described here (*pocytb, pocox1 and poldh*) are 100% identical to this variant. This suggests that the *P. ovale *used by Bray[16,17] was a classic *P. ovale *strain. The data presented here further indicate that *P. ovale *which naturally infects chimpanzees can also infect humans, and that the isolate described here is identical or closely related to the variant isolates sampled in South-East Asia[5,10] and Africa [10]. All five genes sequenced here support the segregation of *P. ovale *into two distinct species.

Molecular typing has clearly demonstrated that *P. knowlesi *infections in Southeast Asia has been misdiagnosed as *P. malariae *and that *P. knowlesi *is much more widespread than previously thought[1,2]. Few data exist on *P. falciparum*, but the recent finding of a new *Plasmodium *in chimpanzees in Gabon closely related to *P. falciparum *and *P. reichenowi *[18,19], show that more studies are needed to fully understand the dynamics of malaria transmission between humans and potential animal reservoirs.

## Competing interests

The authors declare that they have no competing interests.

## Authors' contributions

DT performed the sequencing and carried out the molecular genetic studies in cooperation with JV, and DT and FFA drafted the molecular biology part of the manuscript. EP and JV drafted the medical part of the manuscript. All authors read and approved the final manuscript.

## Informed consent

Written informed consent was obtained from the patient for publication of this case report and accompanying images. A copy of the written consent is available for review by the Editor-in-Chief of this journal.

## Supplementary Material

Additional file 1 **Table S1 Primers used for PCR amplification**. Primers used for PCR amplification. For nested reactions, inner nests use primers with asterisk (*). Concentration (uM) and annealing temperature (Temp) indicated next to sequences. Click here for file
