# Supplementary material for: Variant Plasmodium ovale isolated from a patient infected in Ghana
Source: Malar J. 2011 Jan 22;10:15. doi: 10.1186/1475-2875-10-15 (PMC3037929; doi:10.1186/1475-2875-10-15)
Supplement: Additional file 1 — Table S1 Primers used for PCR amplification. Primers used for PCR amplification. For nested reactions, inner nests use primers with asterisk (*). Concentration (uM) and annealing temperature (Temp) indicated next to sequences. [file 1475-2875-10-15-S1.DOCX]

**Additional files**

Additional file 1

Table S1

Title: Primers used for PCR amplification.

Description: Primers used for PCR amplification. For nested reactions, inner nests use primers with asterisk (*). Concentration (uM) and annealing temperature (Temp) indicated next to sequences.

|  | Forward | Reverse | uM | Temp |
| --- | --- | --- | --- | --- |
| *possrdna* | TCAAAGATTAAGCCATGCAAGTGA | CCTGTTGTTGCCTTAAACTCC | 250 | 55 |
| *possrdna** | TTTTTATAAGGATAACTACGGAAAAGCTGT | TAACCAGACAAATCATATTCACGAAC | 250 | 58 |
| *pocytb* | ATGAATTATTATTCTATTAATTTAG | TGTTTGCTTGGGAGCTGTAATCA | 300 | 50 |
| *pocox1* | CGCCTGACATGGATGGATAATAC | CCATTTAAAGCGTCTGGATAATC | 1000 | 62 |
| *poldh* | GGNTCDGGHATGATHGGAGG | GCCATTTCRATRATDGCAGC | 1000 | 52 |
| *poldh** | TGTDATGGCWTAYTCVAATTGYMARGT | CCATYTTRTTNCCATGWGCWSCDACA | 1000 | 61 |
| *pocysp* | GCCAGTGTAGGTAATATTGAAT | GTATAAAATATCATCATCATCA | 300 | 50 |
